# REXO2 Is an Oligoribonuclease Active in Human Mitochondria

**DOI:** 10.1371/journal.pone.0064670

**Published:** 2013-05-31

**Authors:** Francesco Bruni, Pasqua Gramegna, Jorge M. A. Oliveira, Robert N. Lightowlers, Zofia M. A. Chrzanowska-Lightowlers

**Affiliations:** 1 The Wellcome Trust Centre for Mitochondrial Research, Institute for Ageing and Health, Newcastle University, The Medical School, Newcastle upon Tyne, United Kingdom; 2 The Wellcome Trust Centre for Mitochondrial Research, Institute for Cell and Molecular Biosciences, Newcastle University, The Medical School, Newcastle upon Tyne, United Kingdom; 3 *REQUIMTE*, Department of Drug Sciences, Faculty of Pharmacy, University of Porto, Porto, Portugal; Auburn University, United States of America

## Abstract

The *Escherichia coli* oligoribonuclease, ORN, has a 3′ to 5′ exonuclease activity specific for small oligomers that is essential for cell viability. The human homologue, REXO2, has hitherto been incompletely characterized, with only its *in vitro* ability to degrade small single-stranded RNA and DNA fragments documented. Here we show that the human enzyme has clear dual cellular localization being present both in cytosolic and mitochondrial fractions. Interestingly, the mitochondrial form localizes to both the intermembrane space and the matrix. Depletion of REXO2 by RNA interference causes a strong morphological phenotype in human cells, which show a disorganized network of punctate and granular mitochondria. Lack of REXO2 protein also causes a substantial decrease of mitochondrial nucleic acid content and impaired *de novo* mitochondrial protein synthesis. Our data constitute the first *in vivo* evidence for an oligoribonuclease activity in human mitochondria.

## Introduction

Ribonucleases are enzymes capable of cleaving the phosphodiester bonds between the nucleotide subunits of RNA molecules. Both endo- and exoribonucleases play important roles in the regulation of RNA levels, performing functions in both RNA processing and degradation [1]. All the known exoribonucleases have been divided into six superfamilies based on their sequence, structure and catalytic activity, with the vast majority exhibiting 3′ to 5′ degrading activity [2]. The largest is the superfamily DnaQ-like or DEDD named for the four conserved acidic residues that cluster around the catalytic site of all family members. This distinctive core is essential for binding the divalent metal ions that are required for catalysis [3]. The DnaQ-like domain contains three conserved sequence motifs, namely ExoI, ExoII and ExoIII [4], [5], each containing this distinctive core although the conservation patterns of the three motifs may vary between different subfamilies.

One group of the DEDD superfamily contains oligoribonucleases (oligoRNases) that are essentially specific for small single-stranded RNA fragments of less than five nucleotides in length, although some including human also display DNase activity. An intriguing feature of this group is their high conservation and distribution among the different phyla: they are represented in vertebrates, invertebrates (insects and worms), yeast and some plants [6]. Functional studies of the *orn* gene in *E. coli* have suggested that inactivation gives rise to the deleterious accumulation of short oligoribonucleotides causing cell death, confirming that the enzyme performs a critical role in cell metabolism [7]. Depletion of ORN in *P. aeruginosa* also promotes accumulation of short RNA species, which are able to prime transcription, such that depletion results in global alterations in gene expression [8]. These examples demonstrate that oligoribonucleases perform crucial functions in the final steps of RNA decay and can influence the transcription initiation process in bacteria.

The ORN homologue in *S. cerevisiae*, named YNT20 [9] or REX2p, has been shown to localize to mitochondria and to have a function in the mitochondrial DNA escape pathway that is mediated by YME1, a protease tightly associated with the inner mitochondrial membrane [10]. In contrast to its previous characterization as exclusively mitochondrial, YNT20 is required for the processing of U4, U5L, U5S snRNAs as well as for the maturation of RNA components of both RNase P and 5.8S rRNA in yeast [11] all processes that occur within the nucleus.

Very few human mitochondrial RNases have been fully characterized and none appear to be responsible for degrading very short oligoribonucleotides into monoribonucleotides as the final step of mitochondrial RNA decay process [12]. Here we demonstrate that REXO2 is indeed an active mitochondrial protein, which is also localised to the cytosol. We provide evidence that this enzyme is essential for cell turnover and whose depletion affects mitochondrial structure and function. Surprisingly, we found the majority of REXO2 to be localized in the intermembrane space (IMS) and comment on this observation in our discussion.

## Materials and Methods

### Tissue Culture, and Transfections

Human HeLa cells were cultured (37°C, humidified 5% CO2) in Eagle’s MEM (Sigma) supplemented with 10% (v/v) foetal calf serum (FCS), 1× non-essential amino acids (NEAA) and 2 mM L-glutamine. 143B.206 rho^0^ osteosarcoma cells [13] were propagated in Dulbecco’s modified Eagle’s medium supplemented with 10% (v/v) FCS, 50 µg/ml uridine and 1× NEAA. Flp-InT-Rex-293 cells (HEK293T; Invitrogen) were grown in identical media supplemented with 10 µg/ml Blasticidin^S^ and 100 µg/ml Zeocin (Invitrogen) and transfected as previously described [14]. Post transfection selection was performed with Hygromycin^B^ (100 µg/ml).

Stable transfections were performed using constructs described below and Superfect (Qiagen) using manufacturer’s recommendations.

SiRNA transfections were performed with 60% confluent HeLa cells using Lipofectamine RNAiMAX (Invitrogen) in Optimem-I medium (Gibco) with 25 nM siRNA. Reverse transfections were performed as described by Ovcharenko et al (2005) [15] using approximately 12 000 HEK293 Tcell per cm^2^ using Lipofectamine RNAiMAX in Optimem-I medium (25 nM siRNA). Custom and control non-targetting (NT, Eurogentec ref OR-0030-neg05) duplex siRNAs were purchased pre-annealed from Eurogentec (REXO2 sense 5′-GCAGGCAGAGUAUGAAUUUdTdT; antisense 5′- AAAUUCAUACUCUGCCUGCdTdT.).

All constructs were prepared by PCR using an oligo-dT primed cDNA pool as template. Constructs to facilitate inducible expression of full length C-terminal FLAG-tagged REXO2 were prepared by generating an amplicon from nt 134 to 851 (NM_015523.3) using the following primer pair: FL For 5′- CACACAGGATCCGTCCCGGGTGATGCTAG -3′ and FL FLAG Rev 5′- ACTCGACTCGAGCTACTTATCGTCGTCATCCTTGTAATCCACGGTCTTCTCATTTTCC -3′. The mature, N-terminal truncated untagged REXO2 was amplified using forward primer 5′- CACACAGGATCCACCATGGTCCGCGAAGGTGGC paired with REXO2 no tag rev 5′- ACTCGACTCGAGTCAACTCACGGTCTTCTC. Full length untagged REXO2 was amplified using FL For and REXO2 no tag rev primers. The amplicons and pcDNA5/FRT/TO (Invitrogen) were digested with BamHI/XhoI (sites underlined) and ligated. The GST-fusion construct was made of the mature form of REXO2. The PCR product was generated using the primer pair 5′- CACACAGGATCCGTCCGCGAAGGTGGC and REXO2 rev primer above incorporating a BamHI site in the forward and XhoI site in the reverse primers. The amplicon and vector were digested to allow in-frame fusion of REXO2-GST in pGEX-6P-1 (GE Healthcare).

The constructs derived from pGEX-6P-1 or pcDNA5/FRT/TO were used for transfection of *E. coli* Rosetta pLysS (Merck Biosciences) or HEK293 T cells, respectively. Bacteria were induced, then protein was purified and cleaved from GST using PreScission Protease exactly as previously described for mtRRF [16].

### Mitochondrial Preparation and Subfractionation

For subcellular localization cells were harvested, resuspended in homogenization buffer (HB, 0.6 M Mannitol, 1 mM EGTA, 10 mM Tris-HCl pH 7.4) +0.1% BSA, subjected to standard differential centrifugation with mitochondria being finally pelleted at 11 kg, 10 mins, 4°C and resuspended in HB. The postmitochondrial fraction was retained after centrifugation of the first homogenization. Aliquots of mitochondria were treated with proteinase K (4 µg/100 µg protein) for 30 min at 4°C and either lysed (1% final v/v Triton X-100) or treated with 1 mM PMSF before separation through 15% PAG and transfer to a PVDF membrane. For sub-mitochondrial fractionation, 300µg of mitochondria prepared as described above were then treated with proteinase K (1.5µg) on ice for 30 mins followed by addition of PMSF (5 mM) to generate shaved mitochondria. This was pelleted at 11 kg, 10 mins, 4°C and resuspended in HB (75µl), with 15µl removed as shaved mitochondria. The remainder was pelleted and resuspended in 9 volumes of 10 mM Tris-HCl pH 7.4, divided in half and proteinase K (0.6µg) added to one sample. Both samples were left on ice for 30 mins before addition of PMSF (5 mM) and an equal volume of 1.2 M Mannitol, 2 mM EGTA, 10 mM Tris-HCl pH 7.4. After centrifugation at 12 kg, 10 mins, 4°C, both pellets were resuspended in HB (30µl), and 15µl of each were retained as shaved and unshaved mitoplasts. To prepare the inner membrane fraction, HB (435µl) was added to the proteinase K treated mitoplasts, which were pelleted at 12 kg, pellet was dissolved in 100 mM Na_2_CO_3_ and incubated for 10 mins at 4°C. This was centrifuged at 100 kg for 15 mins at 4°C after which the pellet was resuspended in 2×dissociation buffer (20% (v/v) glycerol, 4% (w/v) SDS, 250 mM Tris-HCl pH 6.8, 100 mM DTT).

### Imaging

For nucleoid and mitochondria morphology imaging, cells were incubated with 3µl/ml PicoGreen (Quant-IT™, Invitrogen) in culture media, for 1 h at 37°C with 5% CO_2_. 5 nM tetramethylrhodamine methyl ester (TMRM^+^, Invitrogen) was added in the last 15 min of incubation and kept throughout washing and imaging in assay buffer containing 135 mM NaCl, 5 mM KCl, 0.4 mM KH_2_PO_4_, 1.3 mM CaCl_2_, 1 mM MgSO_4_, 5.5 mM glucose and 20 mM HEPES (pH 7.4 with NaOH). Imaging acquisition was performed at 63× magnification with an inverted fluorescence microscope (Axiovert 200 M, Carl Zeiss) equipped with FITC and Texas Red filters.

For *in situ* respiratory chain analysis, cells were incubated and assayed in the presence of 25 nM TMRM^+^. Inhibition of respiratory complexes was performed by the following order: 1) Complex V (2µg/ml oligomycin); 2) Complex I (1µM rotenone); and Complex III (2.5µM antimycin A). Full collapse of mitochondrial membrane potential was evoked at the end of experiments by adding uncoupler (1µM FCCP). Fluorescence time-lapse imaging was performed at 30 s intervals to minimize photobleaching, using 10× magnification and the Texas Red filter. 25 nM TMRM^+^ sufficed for ‘quench mode’ imaging in our experimental conditions, allowing the ‘oligomycin null-test’ for forward vs. reverse ATPase operation (increased vs. decreased quenching), and the assessment of depolarization by dequenching upon complex I or III inhibition [17].

### Cell Cycle Analysis

Cell cycle analyses were made on a 3 laser (633 nm, 488 nm and 405 nm) FACSCanto II (Becton Dickinson) with measurements for propidium iodide excitation from the 488 laser and collected using a 585/42 bandpass PMT filter and analysed with FACSDiva 6.1.3 software.

### Northern Blotting

Northern blots were performed as described in [18]. Briefly, aliquots of RNA (5 µg) were electrophoresed through 1.2% agarose under denaturing conditions and transferred to GenescreenPlus membrane (NEN duPont) following the manufacturer’s protocol. Radiolabelled probes were generated using random hexamers on PCR-generated templates corresponding to internal regions of the relevant genes.

High resolution northerns were used to analyse tRNAs. Total RNA (2µg) was denatured in 40% formamide/5 mM EDTA at 65°C, 5 mins before being separated through 15% urea polyacrylamide gels in 1× TBE (89 mM Tris borate, 89 mM boric acid, 2 mM EDTA). RNA was transferred to GenescreenPlus membrane (NEN DuPont) in 0.25× TBE at 150 mA, 15 mins followed by 500 mA for 30 mins after which the membrane was exposed to UV (optimal crosslink, Stratalinker, Stratagene) Hybridisation and probes were as described [19].

### qPCR and Southern Blotting

PCR was used to generate 7S probe using the following primer pairs:- For 5′- CTCAACTATCACACATCAACTG and Rev 5′-AGATACTGCGACATAGGGTG, spanning residues 129 to 16223 of the D-loop [20], [21] and 18S For 5′- AAACGGCTACCACATCCAAG and Rev 5′-GGCCTCACTAAACCATCCAA.

To detect levels and/or depletion of mtDNA the following primer pairs were used that were internal to the gene: ND1 For 5′-AATCGCAATGGCATTCC with ND1 Rev 5′-CGATGGTGAGAGCTAAGG; ND4 For 5′-CCATTCTCCTCCTATCCCTCAAC with ND4 Rev 5′-CACAATCTGATGTTTTGGTTAAACTATATTT; 18S For 5′- GTAACCCGTTGAACCCCATT and 18S Rev 5′- CCATCCAATCGGTAGTAGCG.

### Immunoblotting

Western blots were performed and developed as described in [14] and BlueNative PAGE as in [22] using antibodies as follows : COXI, COX2, NDUFA9, NDUFB8, CV-subunit β, porin (Mitosciences); β-actin, FLAG (Sigma); AIF, S6 (NEB); TOM20 (Santa Cruz); REXO2, GDH custom made and against mature recombinant protein.

### OligoRNase Activity Assays

Oligoribonuclease assays were performed essentially as described by Nguyen LH et al [23] with minor modifications. 100 fmol of single-stranded 5-mer RNA (5′-GAUCG-3′, labelled at 5′ end with Alexa 647®1005 fluorophore; Eurogentec) was incubated with either 50–500 fmol of purified recombinant REXO2 or 0.2–5 µg of total cell or mitochondrial lysates. Incubation was carried out at 37°C, unless otherwise indicated, for 30 mins in nuclease buffer [50 mM HEPES-KOH pH 7.4, 50 mM potassium chloride, 10 mM manganese chloride, 0.01% Triton X-100, 10% glycerol and 0.1 mM DTT]. Reactions were terminated with an equal volume of 100% formamide, heated at 80°C for 3 mins and loaded on a 25% polyacrylamide gel without urea. Immediately after the run, fluorescent products were detected using the STORM 860 PhosphorImager (GE Healthcare Life Sciences) and densitometric analysis was made with Image Quant software (GE Healthcare Life Sciences).

### De Novo Mitochondrial Protein Synthesis

Mitochondrial protein synthesis in cultured cells was performed as described by Chomyn (1996) [24] after addition of emetine and pulsed with [^35^S] methionine/cysteine for 2 hours with a 1 hour chase. Aliquots (50 µg) of total cell protein were separated by 15–20% (w/v) gradient SDS–PAGE. Signals were detected using the Storm 860 PhosphorImager and ImageQuant software.

## Results

### Dual Localization of REXO2 to the Cytosol and Mitochondria

Empirical scanning of the Mitocarta database [25] to identify mitochondrial proteins with predicted ribonuclease activity revealed few candidates. We identified one protein, REXO2 (UniProtKB accession: Q9Y3B8; ref seq NP_056338.2), which appears to be ubiquitously expressed in all tissues and organisms. Various algorithms (MitoProt II, TargetP, PSort and Predotar) gave a very high probability of mitochondrial localization, with a predicted pre-sequence of 25 amino acid (TargetP and PSort).

**Figure 1:**
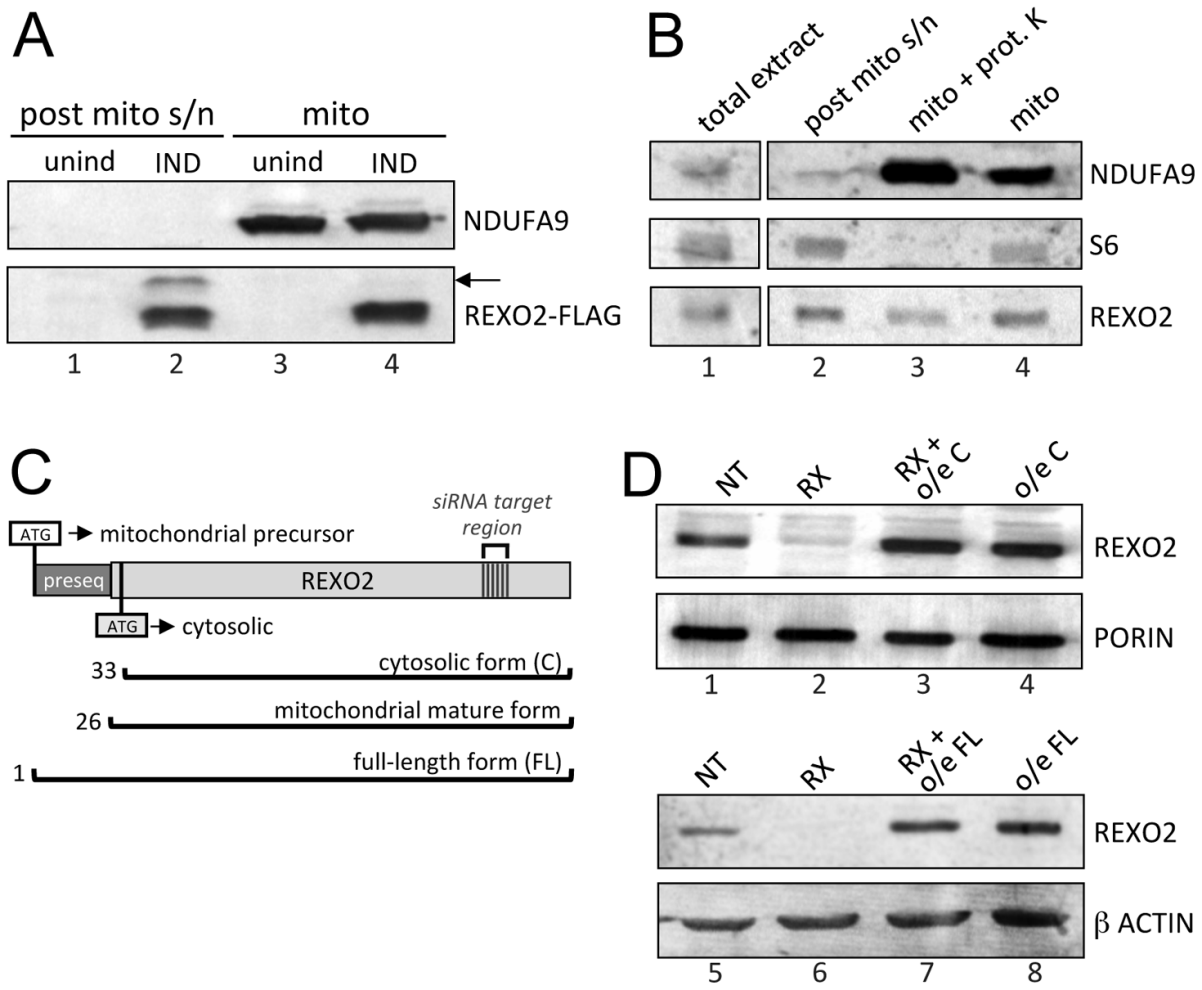
REXO2 is dual localised both to mitochondria and the cytosol. HEK293 cells were engineered to express FLAG tagged REXO2 (panel A lanes 2 and 4). Western blots of post mitochondrial supernatant (40 µg, lanes 1 and 2) and mitochondria (40 µg) were probed with antibodies against a mitochondrial complex I protein (NDUFA9) and FLAG peptide. Arrow indicates species of slower mobility described in text. Panel B is a representative western blot of untransfected HeLa cells (30 µg) probed with antibodies to REXO2, a marker of the cytosol (S6 ribosomal protein) and mitochondrial (NDUFA9). C. Schematic representation of the cDNA encoding REXO2. The two putative translation initiation sites, at amino acid 1 for the full length (FL) and 33 for the cytosolic (C), as well as the predicted cleavage site to generate the mitochondrial mature form following import, are indicated. The position of the siRNA target is indicated, where silent mutations in the cDNA were engineered to render the siRNA unable to bind exogenously expressed transcript. D. HEK293 cell lysates (50 µg) from controls treated with non-targetting (NT) siRNA (lanes 1 and 5), cells depleted of REXO2 (RX) (lanes 2, 3 and 6, 7) or expressing the cDNA encoding the cytosolic form only (C; lanes 3 and 4) or full length (FL; lanes 7 and 8) form of REXO2 were analysed by western blot and probed with antibodies to endogenous REXO2. Antibodies to the mitochondrial outer membrane protein porin, or cytosolic β-actin were used as loading controls.

To experimentally determine the subcellular localization of REXO2, we established a stable human HEK293T cell line inducibly expressing the full length open reading frame carrying a C-terminal FLAG peptide. Following induction cells were fractionated and analysed by western blotting using an anti-FLAG antibody. REXO2 was found localized both to the cytosol and mitochondria (Figure 1A). The post-mitochondrial supernatant contained a second product of lower mobility (indicated by arrow) that was presumed to be the unprocessed mitochondrial precursor. Crucially, this dual localization was also observed for endogenous REXO2 in HeLa cells using custom made anti-REXO2 antibody (Figure 1B). Proteinase K treatment of mitochondria (Figure 1B, lane 3) confirmed that REXO2 is within mitochondria and not simply associated with the outer membrane. Intriguingly, both the endogenous cytosolic (lane 1) and mitochondrial (lanes 3 and 4) forms had similar mobility of approx. 24 kDa, smaller than the predicted Mr of the product encoded by the entire open reading frame (26.8 kDa). Indeed, 24 kDa was similar to the expected size of the mature mitochondrial form after predicted cleavage of 25 N-terminal amino acids (24.4 kDa). The alignment of multiple genomic sequences from several eutherian mammals in the region of the NCBI predicted start site, (Figure S1) shows two in-frame AUGs that are positioned within this region of high conservation. The 5′ proximal AUG has a weak context for translation initiation, potentially encoding the mitochondrial preprotein, while the distal AUG possesses an optimal Kozak consensus facilitating expression of a similar protein without the presequence (23.8 kDa) [26]. An inducible construct lacking the 5′ region of the open reading frame and retaining only the downstream AUG initiation site expressed a product of comparable mobility (Fig 1D lanes 3, 4 *cf.* lane 1) after siRNA depletion of the endogenous protein. This data is consistent with the expression of two isoforms being differentially localized dependent on alternative translation initiation at two in-frame AUGs. We cannot preclude the possibility that only the downstream AUG is used naturally, although this would require mitochondrial localisation and import of only a subset of the polypeptide. Further, the translation initiation complex would need to ignore the 5′ proximal AUG completely. It is also possible that if cleaved, the mature protein could be further trimmed at the N-terminal. Irrespective, the data clearly shows dual cellular localisation of REXO2.

### Sub-mitochondrial Localization of REXO2

**Figure 2:**
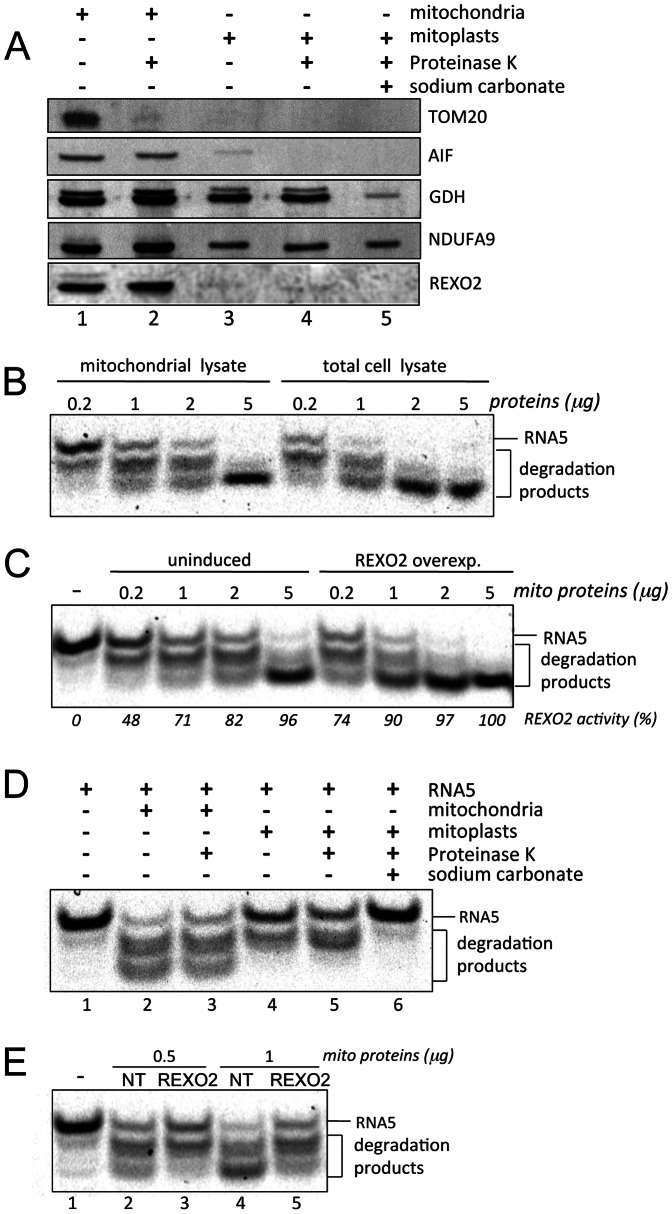
REXO2 localises to two mitochondrial subcompartments where it is active as an oligoRNase. **A.** Mitochondria were prepared from HeLa cells (lane 1), treated with proteinase K (PK) (lane 2) and further subfractionated to give mitoplasts (lane 3), PK shaved mitoplasts (lane 4) and inner membrane fractions (sodium carbonate, lane 5). Aliquots (10 µg) were then subjected to western blotting with antibodies against REXO2 and markers of the mitochondrial outer membrane (TOM20), intermembrane space (AIF), matrix (GDH), inner membrane (NDUFA9). **B.** Lysates were prepared from whole HEK293T cells or mitochondria (increasing protein as indicated). OligoRNase activity was then assayed using 100 fmol of RNA5 substrate as detailed in Materials and Methods. **C.** Mitochondrial lysates were prepared from uninduced HEK293 cells or those expressing REXO2 and activity assayed as in **B.** Maximal activity was defined as when all RNA5 was reduced to the shortest product as shown in far right lane. **D.** Each of the submitochondrial fractions (1 µg) isolated in panel A were then tested for activity (lanes 2–6). Untreated substrate (RNA5) is shown in lane 1. **E.** Mitochondria were extracted from HEK293T cells treated with non-targetting (NT) siRNA (lanes 2, 4) or REXO2 targeted siRNA (lanes 3, 5) and oligoRNase activity assessed in the indicated amounts of lysate.

The intra-mitochondrial localization of REXO2 was then examined by immunoblot of subfractionated HeLa cell organelles (Figure 2A). When intact mitochondria were treated with proteinase K, REXO2 remained intact whereas TOM20, a marker of the outer mitochondrial membrane (OMM), was lost (Figure 2A, lanes 1 and 2). Following OMM disruption, by hypotonic shock to release soluble intermembrane space (IMS) proteins, REXO2 levels dropped dramatically (Figure 2A, lane 3) as did those of the IMS marker apoptosis-inducing factor (AIF). In contrast, glutamate dehydrogenase (GDH), a mitochondrial matrix marker, remained. Proteinase K treatment of these mitoplasts did not further reduce REXO2 signal whilst AIF was no longer detectable (Figure 2A, lane 4) consistent with a fraction of REXO2 being present in the matrix. Inner mitochondrial membrane (IMM) proteins were prepared by sodium carbonate treatment of mitoplasts. In this fraction (Figure 2A, lane 5), REXO2 was undetectable unlike an IMM marker NDUFA9, a member of Complex I. These data infer the majority, but not all, of the mitochondrial REXO2 is present in the IMS.

### Mitochondrial REXO2 is an Active oligoRNase

The ribonuclease activity of REXO2 was tested with various nucleic acid templates but showed a clear preference for oligoRNAs was observed. All subsequent experiments used a single-stranded 5-mer RNA, 5′ labelled with ALEXA 647, as substrate (RNA5). Initial assays used a recombinant version of the mature REXO2 Purified, soluble REXO2 was incubated with substrate at 37°C and 50°C. Robust activity was observed at 37°C with activity retained at 50°C (Figure S2). Mitochondrial and total protein extracts were tested using identical experimental conditions. OligoRNase activity was clearly present in the mitochondrial fraction (Figure 2B). Total cell extract produced greater substrate degradation as expected since both the mitochondrial and cytosolic enzymes will contribute (Figure 2B). Bacterial ORN retains activity at temperatures that typically inactivate the majority of RNases [27]. We therefore tested the mitochondrial fraction for similar heat resistance. Reminiscent of the bacterial enzyme, oligoRNase activity was still detectable at 65°C but retained only minimal activity at 80°C (data not shown).

Is REXO2 responsible for this oligoRNase activity in human mitochondria? To address this, we tested mitochondrial extracts from HEK293T cells overexpressing REXO2 and uninduced controls. Consistent with the hypothesis, the former showed higher levels of robust oligoRNase activity (Figure 2C).

We subsequently tested all the submitochondrial fractions for RNase activity. The activity (Figure 2D) paralleled the distribution of REXO2 observed by western blotting (Figure 2A), in particular showing a greater activity in the whole organelle compared to the mitoplast (Figure 2D, Lane 3 cf lane 5) consistent with greater levels of the enzyme in the IMS than matrix. However, proteinase K treated mitoplasts did retain significant oligoRNase activity confirming that REXO2 is also active in the mitochondrial matrix. To extend the correlative evidence, mitochondria extracted from REXO2 depleted samples were tested for oligoRNase activity and a decrease in activity was confirmed (Figure 2E).

**Figure 3:**
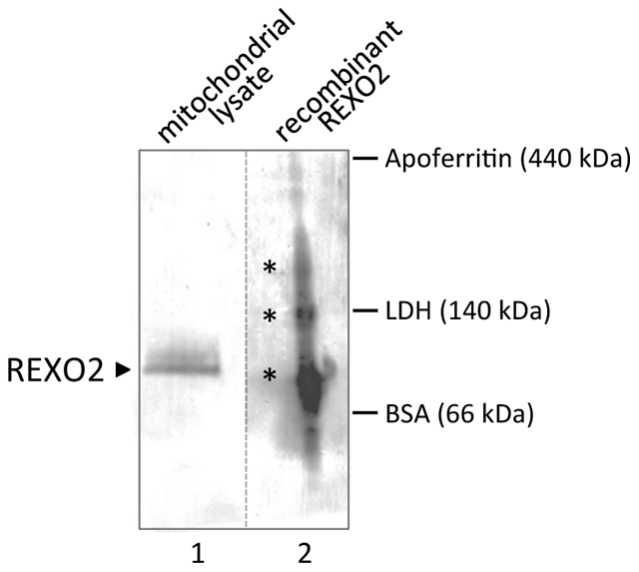
Native mitochondrial REXO2 is present as a tetramer. Purified recombinant REXO2 (2 µg, lane 2) representing the matured mitochondrial form, together with mitochondrial lysate (100 µg, lane 1) from HeLa cells were separated through a 5%-18% gradient blue native polyacrylamide gel. Post-transfer the western blot was interrogated with antibodies against REXO2. Multimeric forms of recombinant REXO2 are indicated by * and the positions of molecular weight markers are given. The dashed line is used to indicate that lanes 1 and 2 required 2 different exposure times.

*Is the enzyme present as a complex ? E. coli* ORN has been described as a dimer based on its chromatographic profile [6]. When recombinant cytosolic REXO2 was analysed by the same approach, it eluted as a single predicted mass of ∼90 kDa [23]. The authors suggested that this represented either a slowly migrating rod shaped structure, or a tetrameric complex [23]. In contrast to this chromatographic approach, we chose to identify the conformation of the mitochondrial form via Blue Native PAGE using mitochondrial lysate and on bacterially expressed purified recombinant mature REXO2. The mitochondrial lysate gave rise to a single 90–100 kDa complex (Figure 3) consistent with REXO2 being present as a homotetramer with four mature monomers of 24.4 kDa. Our untagged recombinant REXO2 also migrated as a homotetramer with some evidence of larger multimeric complexes, possibly the consequence of high concentrations of purified sample. More importantly, our data demonstrate the native mitochondrial enzyme appears to present in a homotetrameric complex.

### Loss of REXO2 Affects Cell Growth and Morphology

**Figure 4:**
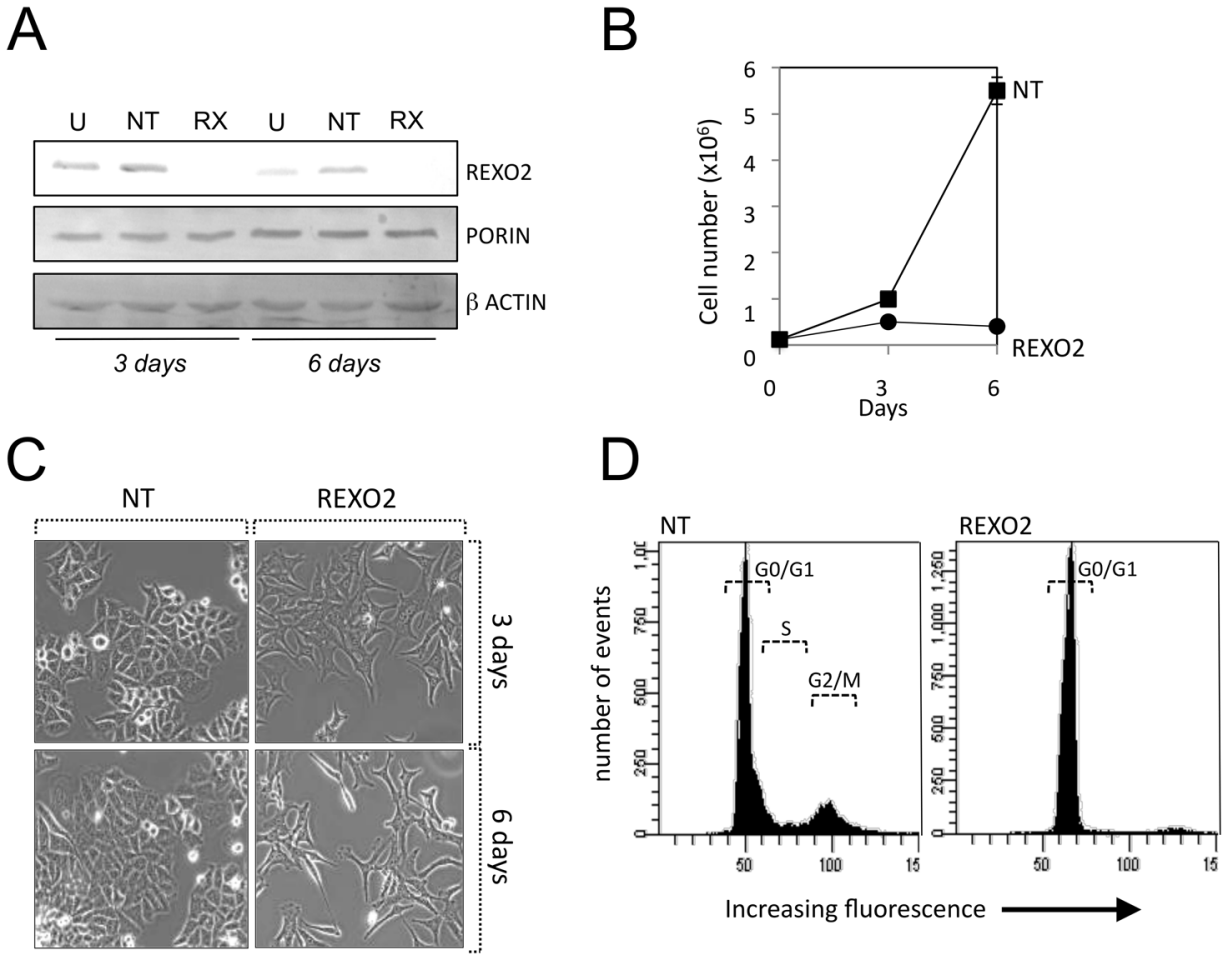
Loss of REXO2 affects cell growth and morphology. **A.** HeLa cells were treated with non-targetting (NT) or REXO2 (RX) directed siRNAs for 3 or 6 days and cell lysates prepared from these and untransfected (U) controls (20 µg). REXO2 depletion was analysed by western blot using antibodies against REXO2 with porin and β-actin acting as controls. Cell growth (panel **B**) and morphology (panel **C**) were monitored over the same time period. Cell cycle status was analysed by propidium iodide staining and FACS (panel **D**)**.** For each reading 10,000 events were sampled.

ORN is required for cell viability in *E. coli* [7]. Since human REXO2 is almost 50% identical to its bacterial counterpart [5] we assessed whether REXO2 is also essential for human cells. Five different siRNAs independently and specifically caused depletion of REXO2 protein in several human cell lines (Figures 1, 2, 4 and data not shown). Those giving over 95% depletion compared to control cells treated with a non-targetting (NT) siRNA were used in all experiments reported in this manuscript. Growth rate was reduced over the first 3 days of REXO2 depletion in HeLa cells after which cells failed to proliferate and displayed a distinctive stellate morphology compared to control cells (Figures 4B and C). To clarify this observation we analysed cell cycle status of control and depleted cells (Figure 4D). Curiously almost the entire population of REXO2-depleted cells was stalled in G0/G1 phase compared to control cells distributed across all phases, suggesting that cells lacking REXO2 become quiescent rather than die, after 6 days depletion.

Rho^0^ cells lack mitochondrial DNA, consequently are devoid of mt-RNA and therefore may be expected to be unaffected if proteins involved in mitochondrial gene expression are depleted. In contrast if proteins are crucial for other cell processes, then rho^0^ cells should be severely affected upon their depletion. Since REXO2 is present in both the cytosol and mitochondria we aimed to dissect the importance of REXO2 in each compartment by depleting human osteosarcoma rho^0^ cells of REXO2. Interestingly, although REXO2 is present in 143B Rho^0^ cells, siRNA mediated depletion of the protein had no significant effect on cell growth (Figure S3) in contrast to parental 143B Rho^+^ cells, HeLa and HEK293 cells all of which showed markedly decreased cell division. This data strongly suggests that REXO2 plays an important role in mitochondrial gene expression, which in turn exerts a level of control of cell division in wild type cells.

### REXO2 is Important in Maintaining Mitochondrial Structure and Function

**Figure 5:**
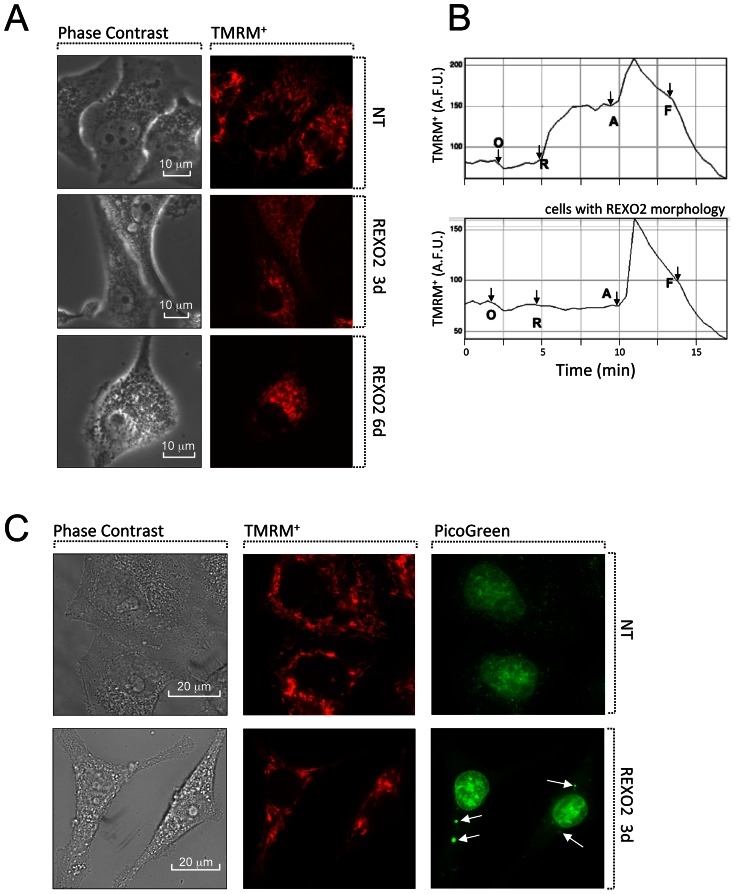
Loss of REXO2 affected mitochondrial morphology and mtDNA distribution. **A.** Representative images of HeLa cells treated with non-targetting (NT) or REXO2 directed siRNAs for 3 and 6 days. Stellate morphology in REXO2 depleted cells was associated with punctate, yet polarized (TMRM^+^ loaded) mitochondria. **B.** Representative time-lapse recordings of TMRM^+^ fluorescence (A.F.U, arbitrary fluorescence units) at single cell resolution, indicative of changes in mitochondrial membrane potential upon sequential complex inhibition with oligomycin (O, 2 µg/ml), rotenone (R, 1 µM), antimycin (A, 2.5 µM), and uncoupler (F, 1 µM FCCP). Cells with stellate REXO2 morphology were associated with undetectable rotenone depolarization. **C.** Representative images of REXO2 depleted cells showing few and abnormally enlarged mitochondrial nucleoids labelled with PicoGreen, in comparison with the normal abundant small nucleoids in NT controls. White arrows indicate the larger nucleoids present in REXO2 depleted cells.

Since the data from REXO2 depleted rho^0^ cells indicates an important role in mitochondrial function, we analysed various mitochondrial parameters following REXO2 depletion. Changes to mitochondrial morphology were analysed in HeLa cells with the lipophilic cationic dye TMRM^+^. It has been established that the form of the mitochondrial network varies with the different stages of the cell cycle with the reticulum being more characteristic of G1 and fragmentation of S phase [28], [29]. It was interesting to note therefore that in contrast to the normal reticulum of actively growing control cells, cells depleted of REXO2 and confirmed as G1/G0 arrested led to the punctate and granular mitochondria associated with S phase, and with mitochondrial dysfunction [30], [31] (Figure 5A).

Single cell measurements were made in cells loaded with TMRM^+^ in quench mode (25nM; a condition in which mitochondrial depolarization increases fluorescence), and sequentially incubated with three respiratory chain inhibitors (oligomycin, rotenone and antimycin) and then uncoupler FCCP (Figure 5B). Both control and REXO2 depleted cells (3 days) showed a modest hyperpolarization to oligomycin, demonstrating that their mitochondria are still net ATP generators (oligomycin null test). However, while control cells showed characteristic double depolarization after the addition of rotenone and antimycin, respectively, REXO2 depleted cells with a stellate morphology were insensitive to rotenone, suggesting a reduced contribution of complex I to mitochondrial membrane potential when compared with control cells. Potential is not being held by ATP synthase reversal (no oligomycin depolarization), thus suggesting a modest rather than severe respiratory chain impairment after 3 days of REXO2 depletion. The status of the mitochondrial membrane potential consistently correlated with the stellate morphology and indicated that loss of REXO2 impairs respiratory chain function.

Both control and depleted cells were also stained using PicoGreen to visualise the non-supercoiled DNA in the nucleus and mitochondrial nucleoids. Interestingly, cells lacking REXO2 appeared to have fewer but larger nucleoids compared to control cells (Figure 5C).

### REXO2 Depletion Severely Affects Mitochondrial Nucleic Acid Content

**Figure 6:**
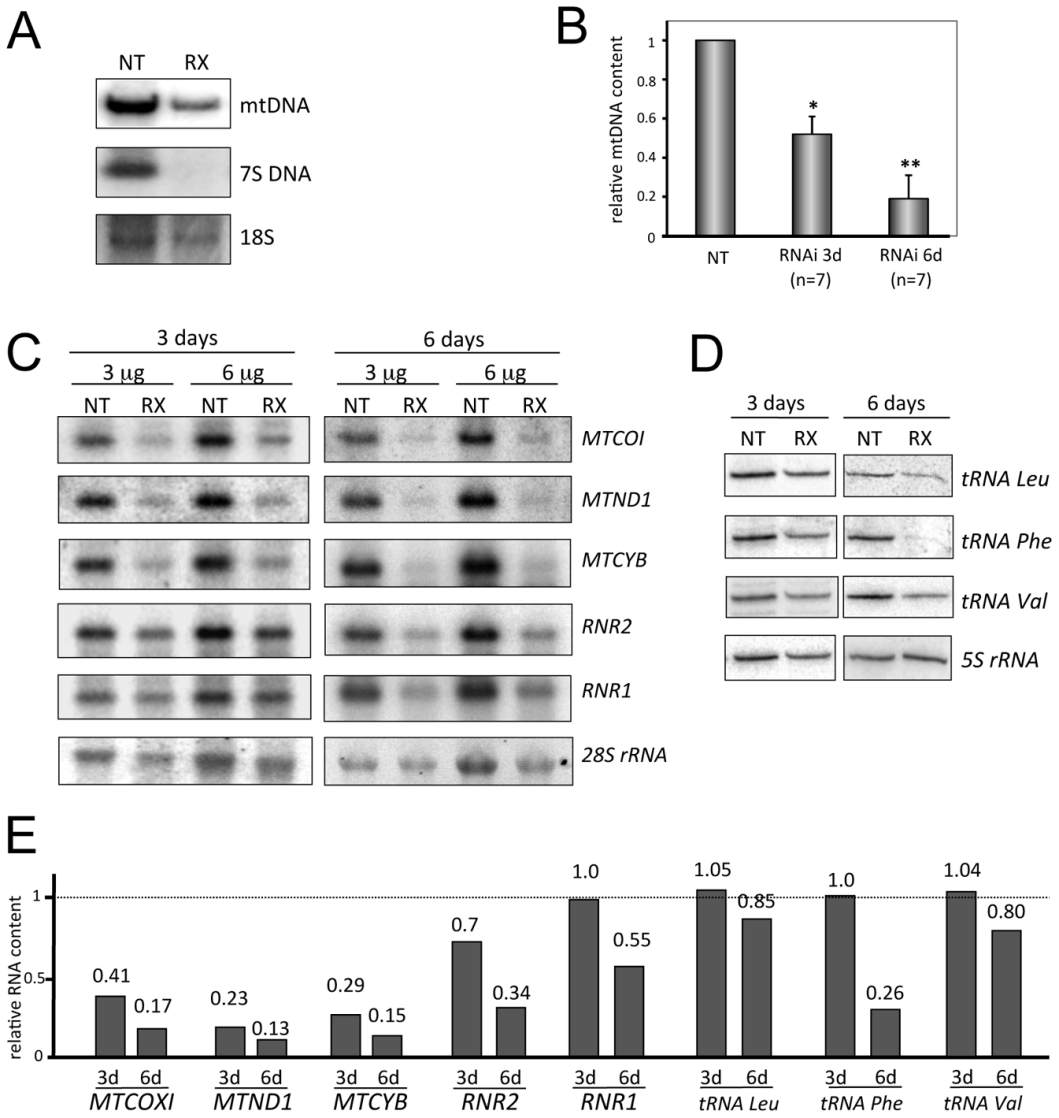
Loss of REXO2 affects mtDNA and mtRNA levels. **A.** HeLa cells were treated with non-targetting (NT) or REXO2 (RX) directed siRNAs for 6 days and DNA prepared. Samples (5 µg) were BamHI digested and separated through a 0.7% agarose gel. The subsequent Southern blot was probed to reveal levels of mtDNA, 7S DNA and nuclear encoded 18S. Quantitative PCR analysis is presented (panel **B).** RNA was extracted and analysed from HeLa cells treated over 3 and 6 days as for **A** above. Northern blot analysis was performed (panel **C**) to investigate changes to mt-mRNA, mt-rRNA levels using 28S as a loading control, whilst high resolution northerns (2 µg RNA/sample) were used to determine changes to mt-tRNA levels (panel **D**)**.** Quantitative analysis of data presented in panels **C** and **D** is given in panel **E**.

To evaluate whether the PicoGreen staining represented mtDNA depletion or an alteration in mtDNA distribution or conformation, we used two approaches. First, quantitative PCR was used to amplify mtDNA corresponding to *MTND1* and *MTND4* genes relative to the nuclear 18S rRNA gene. For both *MTND1* and *MTND4* REXO2 depletion caused a respective decrease of 50% (±7%) and 65% (±10%) after 3 days and 79% (±16%) and 86% (±7%) after 6 days. The lack of a significant difference between levels of the two amplicons, suggests that REXO2 depletion did not cause deletions but only depletion of mtDNA. Southern blotting confirmed the depletion of mtDNA and unexpectedly revealed the apparent disappearance of mitochondrial 7S DNA in REXO2 depleted cells (Figure 6A).

Northern blots were used to assess whether the observed mtDNA decrease affected the steady state levels of mitochondrial RNAs. After 3 days REXO2 depletion mt-mRNAs were most affected of the 3 transcript types, with only a modest decrease in 16S rRNA (*RNR2*) and little variation in 12S rRNA (*RNR1*) or mt-tRNA levels (Figure 6C, D and E) using 28S signal to correct for loading. Six days treatment led to a reduction in levels of all mitochondrial RNA species with mt-mRNAs being the most affected (Figure 6C, D and E). This data is consistent with global mitochondrial transcription being affected, as the stability of mt-rRNAs and mt-tRNAs are far greater than mt-mRNA [32].

### Reduction of Mitochondrial RNAs Perturbs de Novo Synthesis and Steady State Level of Mitochondrial Polypeptides in REXO2 Depleted Cells

Both mitochondrial DNA and RNA levels appear to decrease as a consequence of REXO2 depletion. We evaluated the consequences of this on intra-organellar protein synthesis.

**Figure 7:**
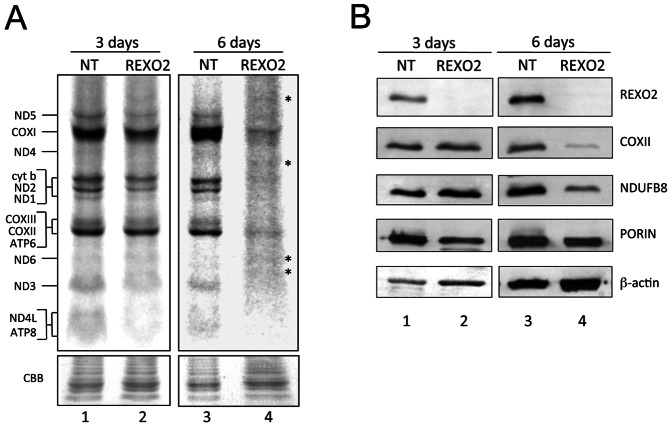
Loss of REXO2 perturbs de novo synthesis and steady state levels of mitochondrially encoded proteins. HeLa cells were treated with non-targetting (NT) or REXO2 siRNA for 3 and 6 days after which cytosolic translation was inhibited and mitochondrial protein synthesis analysed by ^35^S met/cys incorporation (2 hr followed by a 1 hr chase). Cell lysate (50 µg) was separated through 15–20% PAG. Post fixation and drying the signal was visualised by STORM 860 PhosphorImaging and ImageQuant software (GE Healthcare Life Sciences). The gel was rehydrated and stained with Coomassie blue (CBB) to confirm equal loading. Signals were ascribed following migration patterns described in [24] and aberrant products in lane 4 are indicated by asterisks. **B.** Steady state levels of mitochondrial proteins from cells treated as in (**A**) were analysed by western blot of cell lysates (25 µg). Antibodies were used against REXO2, a mtDNA-encoded protein (COXII), a nuclear encoded complex I protein (NDUFB8), a nuclear encoded mitochondrial protein (porin) and a cytosolic control (β-actin).

*De novo* synthesis of mtDNA encoded products was assessed by whole cell metabolic labelling. Mitochondrial protein synthesis was partially affected after three days of REXO2 depletion and severely affected after six days treatment (Figure 7A), when aberrantly migrating polypeptides could also be observed. This pattern was consistent with the observed reduction of mitochondrial RNAs that are required for mitochondrial translation. We then measured the steady-state levels of COX2 (Complex IV) and NDUFB8 (Complex I). There was little evidence of change after 3 days but by six days of REXO2 siRNA treatment these subunits showed a robust decrease in steady state levels when corrected for β-actin levels (Figure 7B).

## Discussion

Correct maintenance of mitochondrial RNA level is crucial for organellar metabolism. The steady state levels are regulated by balancing transcription against RNA stability and turnover (reviewed in [33]–[36]. Degradation not only contributes to maintaining mt-RNA levels but also eliminates by-products of both processing and aberrant transcripts [37]. In eubacteria this process requires the concerted action of exo- and endo-ribonucleases, giving rise to small oligoribonucleotides that have to be completely degraded. This last step is essential to recycle and make available monoribonucleotides for transcription.

Given the essential nature of RNA decay, it has been a challenging endeavour to identify enzymatic activities responsible for RNA metabolism in mammalian mitochondria, and very few RNases have been characterized thus far [12], [38]. Further, none of these enzymes has been demonstrated to be specific for short oligoribonucleotides. In this paper we report that REXO2 is such a protein. First, we demonstrate that human REXO2 is a mitochondrial protein as well as being found in the cytosol. Interestingly, although there is a single major transcript it contains two in-frame start codons which are highly conserved. Although we have not shown unequivocally how the dual targeting originates, data was consistent with translation initiation from the upstream AUG generating a protein with a cleavable presequence that is destined for the mitochondrion, whilst use of the downstream start site generates the cytosolic form of REXO2. Such a mechanism would be shared by other mitochondrial proteins with a dual localization [39], [40] including two RNases; RNase Z, responsible for the removal of 3′ extensions from tRNA precursors [41], and RNase H1, which has a putative role during mtDNA replication [42].

We demonstrate that mitochondrially localized REXO2 possesses a 3′-5′ exonuclease activity specific for small RNA oligomers (Figure 2) that could be generated during mitochondrial replication or transcription. The machineries responsible for these two fundamental processes are contained within nucleoids in the mitochondrial matrix [43] and the size and distribution of these features is altered upon REXO2 depletion. We found dual submitochondrial localization of REXO2. It was detectable in both the mitochondrial matrix and the IMS. This unusual dual localization is reminiscent of another mitochondrial RNase, PNPase. The latter has been localized to the mitochondrial matrix [44] and the IMS [45] mediating translocation of several RNAs into mitochondria [46]. PNPase has also been co-purified with the matrix helicase SUV3; an enzyme with roles in mt-RNA turnover/surveillance [37] and mtDNA maintenance [47]. Moreover, PNPase and SUV3 are able *in vitro* to form an active heteropentameric complex that efficiently degrades structured RNAs [48] interestingly leaving oligoRNAs of 4 nucleotides in length [49], which may be *in vivo* substrates for REXO2. Our data demonstrate that loss of REXO2 affects mitochondrial structure and function, causing a disorganized mitochondrial network with evident punctate and granular mitochondria. Notably, an identical alteration in the mitochondrial reticulum structure was found previously in human cells by depleting SUV3 [47] or PNPase [45]. REXO2 depletion also has a negative effect on both replication and transcription in mitochondria, causing a robust depletion of mtDNA and of mtRNAs. This result is consistent with a role for REXO2 as mitochondrial nucleotide scavenger, since both mtDNA replication and transcription would benefit from REXO2-mediated recycling of mono-ribonucleotides.

An intriguing finding was the loss of mitochondrial 7S DNA concomitant with the formation of fewer but larger nucleoids in REXO2 depleted cells. A similar correlation has been found by Holt and colleagues in cells over-expressing the accessory subunit of mitochondrial DNA polymerase γ,POLGβ [50]. One explanation might be that through a role in recycling the mono-ribonucleotides necessary for RNA primer synthesis in the D-loop, REXO2 depletion could negatively affect the synthesis of 7S DNA. It has been hypothesised that 7S DNA facilitates the recruitment of ATAD3p, which can mediate nucleoid formation and division [51]; this, together with the observation of a strong depletion of mtDNA, could explain the presence of few but large nucleoids in cells lacking REXO2.

What is the function of REXO2 in the cytosol ? Our work has not addressed this question directly. The absence of any notable growth phenotype on depletion of REXO2 from rho^0^ cells implies that it does not have an essential function and is not as crucial to the cell as the mitochondrial form. However, it is possible that the cytosolic function is maintained even with the very low level of REXO2 present after siRNA depletion or there is another enzyme capable of performing this function.

In conclusion, our data provided the first evidence for an oligoribonuclease activity in human mitochondria. Our study demonstrates that REXO2, shares similarities with its bacterial counterpart, ORN, is important for cell viability, and plays a role in mtDNA and mtRNA maintenance. Future work will clarify its function in the IMS and establish interactions with other mitochondrial RNases.

## Supporting Information

Figure S1 **REXO2 has conserved two AUG translation initiation sites.** Alignment of five eutherian mammalian sequences for REXO2 using ClustalW2 shows extremely high levels of conservation. This is especially evident in the regions of the two in frame AUG start codons (boxes I and II) and the Kozak consensus at the downstream AUG (box II). The predicted starting position of the matured mitochondrial sequence is indicated.
(TIF)

Figure S2 **Recombinant REXO2 preparation and oligoRNase activity. A.** Purified recombinant REXO2 was separated by 12% PAGE and the gel stained with Coomassie blue to confirm purity and concentration (Lanes 1–5; 100 ng, 500 ng, 1 µg, 2.5 µg, 5 µg). **B.** OligoRNase activity was then assayed using 100 fmol of substrate (lane 1) with increasing amounts of protein at 37°C (lanes 2–4) and 50°C (lanes 5–7).
(TIF)

Figure S3 **REXO2 depletion has no effect on growth of rho^0^ cells.** 143B rho^0^ cells were treated with non-targetting (NT) or REXO2 (RX) siRNA for 3 days. Initial number of cells seeded is indicated by the dotted line on the graph (**A**) and the increase in cell number was analysed, as was cell morphology (**B**)**.** A western blot of 143B rho^0^ cells treated with non-targetting (NT) or REXO2 (RX) siRNA for 3 days is presented in Panel **C.** Cell lysates (40 µg) were separated by 12% SDS PAGE and the blot interrogated with antibodies to endogenous REXO2 and β actin as a loading control.
(TIF)
