# Supplementary figures and images for: REXO2 Is an Oligoribonuclease Active in Human Mitochondria
Source: PLoS One. 2013 May 31;8(5):e64670. doi: 10.1371/journal.pone.0064670 (PMC3669425; doi:10.1371/journal.pone.0064670)

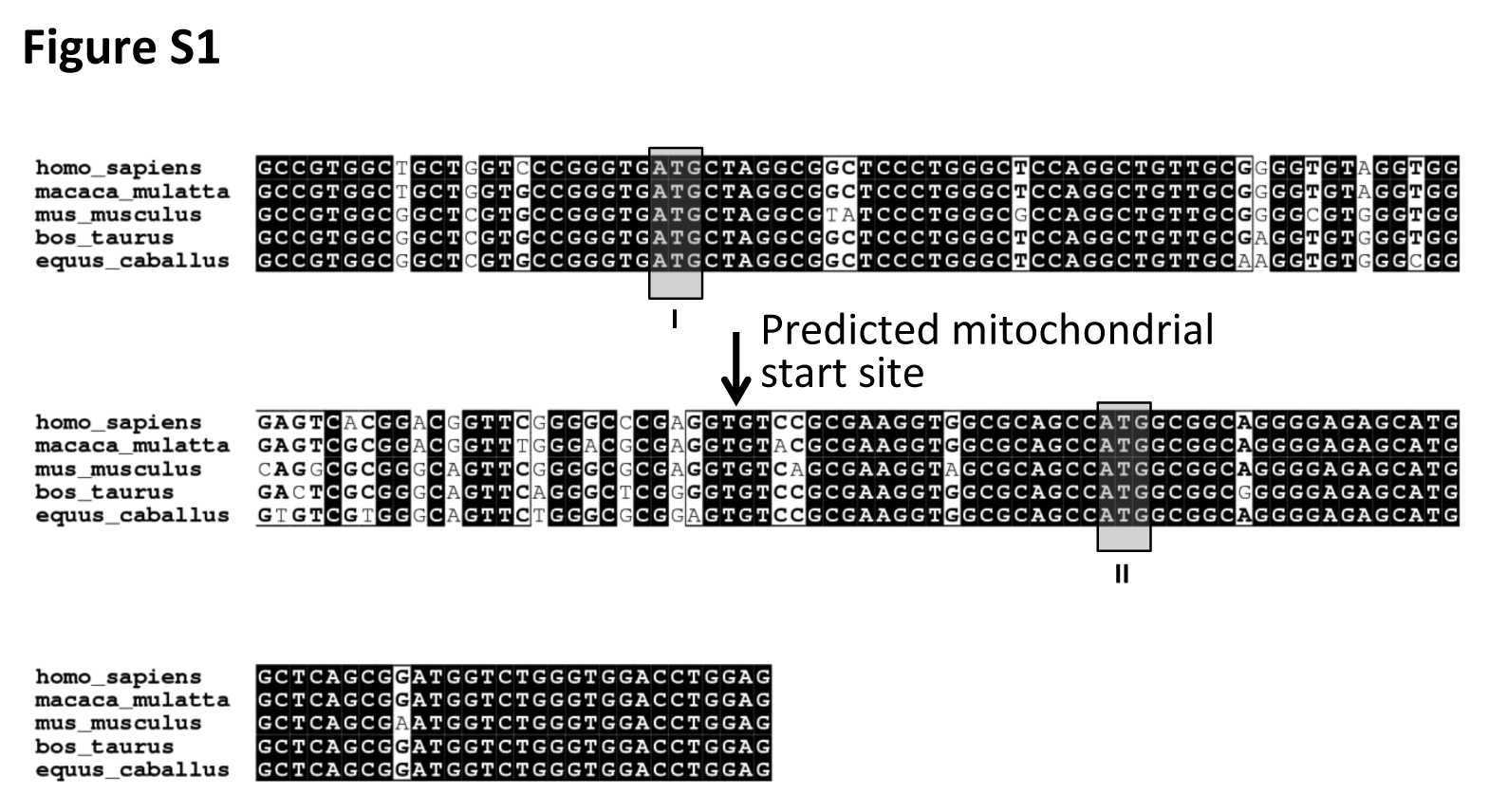

Supplement: Figure S1 — REXO2 has conserved two AUG translation initiation sites. Alignment of five eutherian mammalian sequences for REXO2 using ClustalW2 shows extremely high levels of conservation. This is especially evident in the regions of the two in frame AUG start codons (boxes I and II) and the Kozak consensus at the downstream AUG (box II). The predicted starting position of the matured mitochondrial sequence is indicated. (TIF) [file pone.0064670.s001.tif]

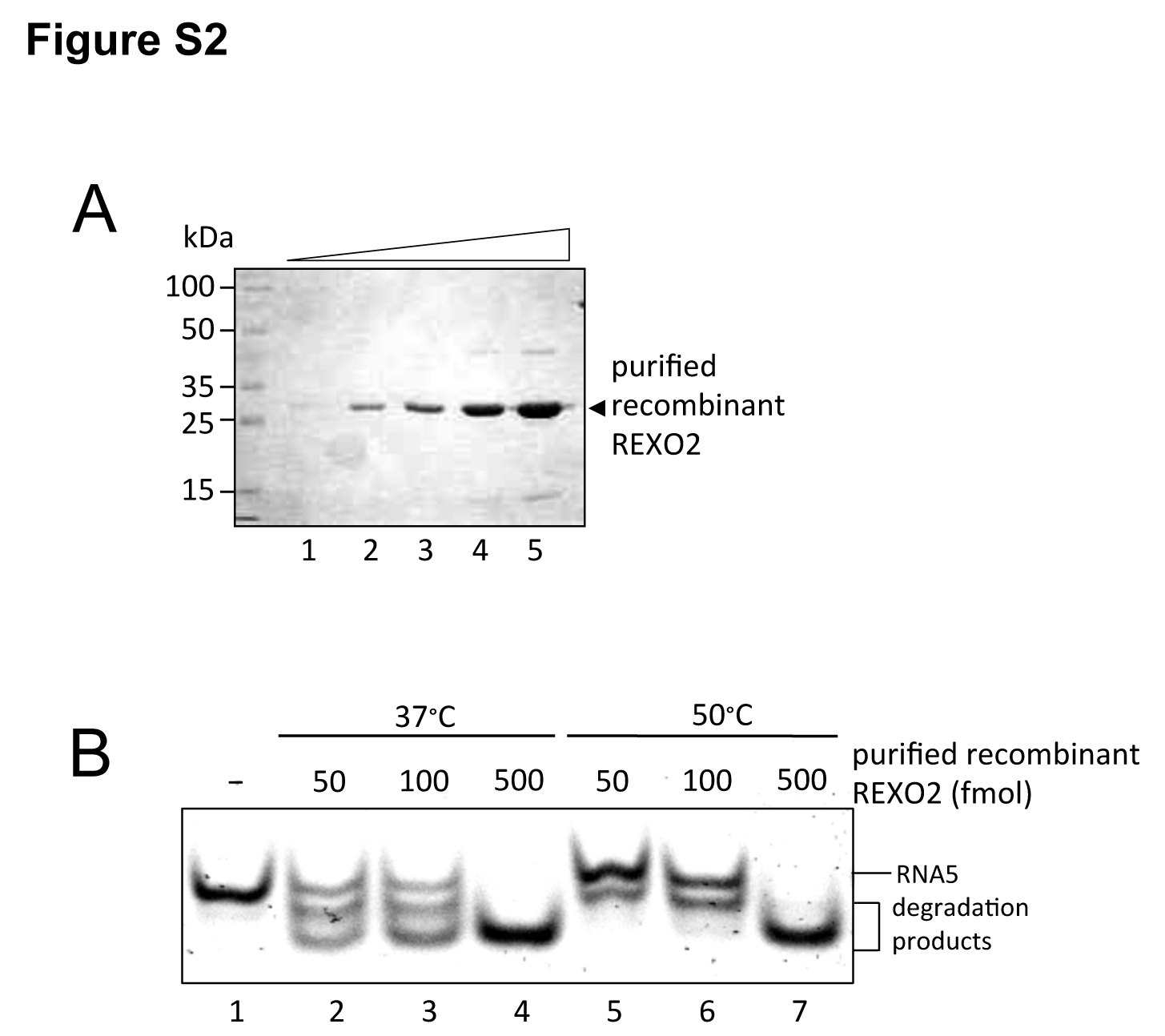

Supplement: Figure S2 — Recombinant REXO2 preparation and oligoRNase activity. A. Purified recombinant REXO2 was separated by 12% PAGE and the gel stained with Coomassie blue to confirm purity and concentration (Lanes 1–5; 100 ng, 500 ng, 1 µg, 2.5 µg, 5 µg). B. OligoRNase activity was then assayed using 100 fmol of substrate (lane 1) with increasing amounts of protein at 37°C (lanes 2–4) and 50°C (lanes 5–7). (TIF) [file pone.0064670.s002.tif]

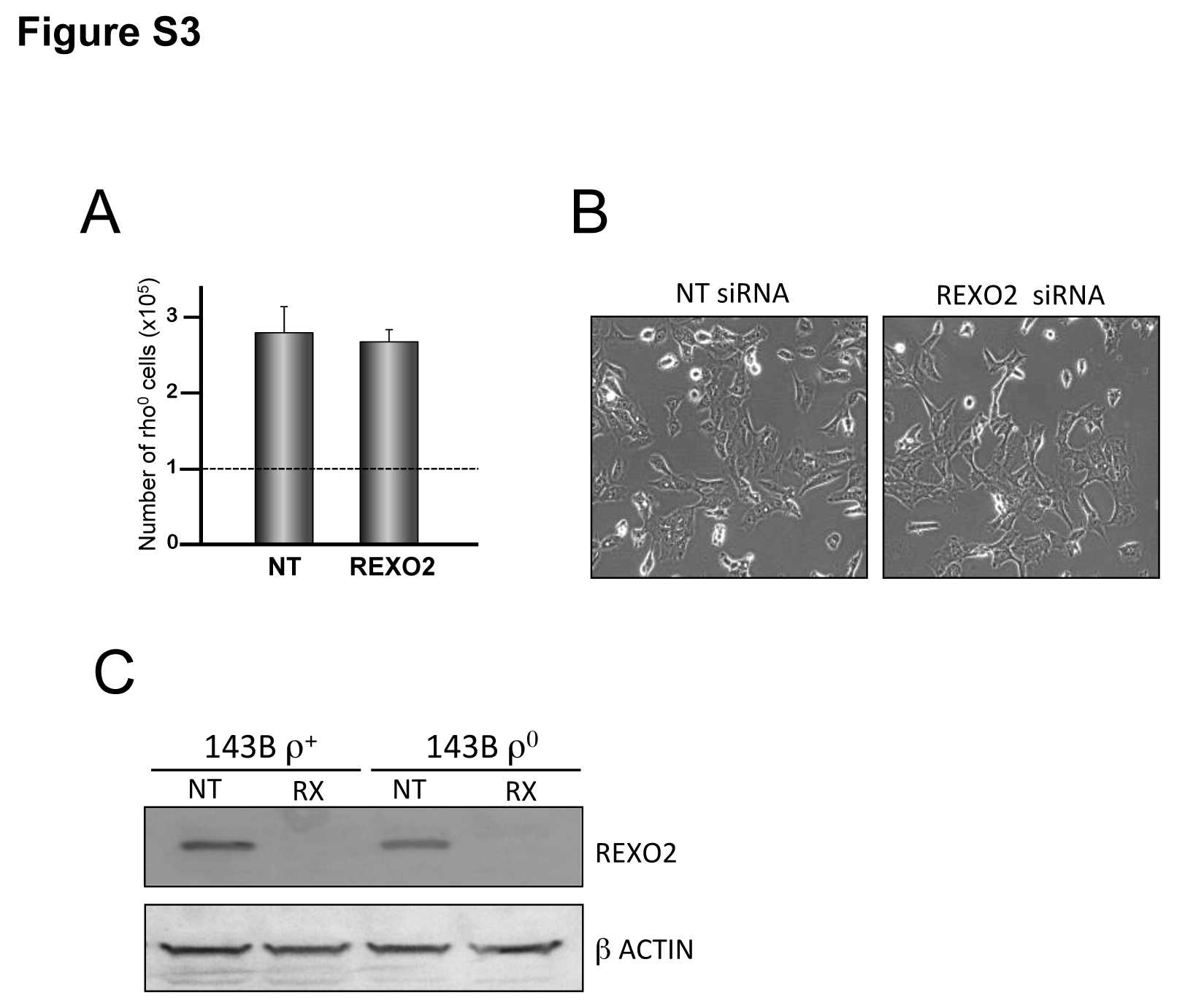

Supplement: Figure S3 — REXO2 depletion has no effect on growth of rho0 cells. 143B rho0 cells were treated with non-targetting (NT) or REXO2 (RX) siRNA for 3 days. Initial number of cells seeded is indicated by the dotted line on the graph (A) and the increase in cell number was analysed, as was cell morphology (B). A western blot of 143B rho0 cells treated with non-targetting (NT) or REXO2 (RX) siRNA for 3 days is presented in Panel C. Cell lysates (40 µg) were separated by 12% SDS PAGE and the blot interrogated with antibodies to endogenous REXO2 and β actin as a loading control. (TIF) [file pone.0064670.s003.tif]
